# The impact of the flagellar protein gene *fliK* on *Helicobacter pylori* biofilm formation

**DOI:** 10.1128/msphere.00018-25

**Published:** 2025-03-21

**Authors:** Hongjin Tao, Wangjingyi Zhang, Jing Liu, Yu Zhou, Gangshi Wang

**Affiliations:** 1 Medical School of Chinese PLA 104607 , Beijing, China; 2 Department of Gastroenterology, The Second Medical Center & National Clinical Research Center for Geriatric Diseases, Chinese PLA General Hospital, Beijing, China; 3 Institute of Geriatrics, National Clinical Research Center of Geriatrics Disease, Second Medical Center, Chinese PLA General Hospital, Beijing, China; 4 Department of Laboratory Medicine, Second Medical Center, Chinese PLA General Hospital, Beijing, China; Vanderbilt University Medical Center, Nashville, Tennessee, USA

**Keywords:** *Helicobacter pylori*, biofilm, gene knockout, *fliK*

## Abstract

The biofilm structure of *Helicobacter pylori* is known to enhance its capabilities for antimicrobial resistance. This study aims to investigate the role of the flagellar hook length control protein gene *fliK* in the biofilm formation of *H. pylori*. Homologous recombination was employed to knock out the *fliK* gene in the *H. pylori* NCTC 11637 strain. The flagella of *H. pylori* were observed using transmission electron microscopy (TEM), whereas *H. pylori* motility and growth were examined through semi-solid agar assays and growth curve analyses, respectively. The bacterial biofilm and its constituents were visualized utilizing fluorescence confocal microscopy. Assessments of *H. pylori* adhesion to gastric mucosal cells, its vacuolar toxicity, and antibiotic resistance were evaluated using co-culture experiments and E-test methods. The *fliK* gene was successfully knocked out in *H. pylori* NCTC 11637. The Δ*fliK* mutant exhibited polyhook structures or lacked typical flagellar morphology, reduced mobility, and a slower bacterial growth rate compared with the wild-type strain. Fluorescence confocal microscopy revealed a decrease in the thickness of the biofilm formed by the Δ*fliK* strain, along with reductions in polysaccharide and DNA components. The deletion of *fliK* did not affect vacuolar toxicity or antibiotic resistance but did reduce the adhesive capacity of the bacterium to gastric mucosal cells. The deletion of the *fliK* gene significantly impairs *H. pylori* biofilm formation, leading to substantial decreases in biofilm components, bacterial growth, and adhesion capabilities. These findings underscore the importance of *fliK* in the pathogenicity of *H. pylori*.

**IMPORTANCE:**

The increasing antibiotic resistance of *Helicobacter pylori* has emerged as a global health concern, with biofilm formation serving as a crucial mechanism underlying this resistance. This study investigates the role of the *fliK* gene, which encodes the flagellar hook length control protein, in *H. pylori* biofilm formation. Furthermore, we examined the influence of *fliK* on *H. pylori* growth, motility, and cellular adhesion capabilities. Our findings elucidate the molecular mechanisms governing *H. pylori* biofilm formation and suggest potential therapeutic strategies for addressing *H. pylori* antibiotic resistance.

## INTRODUCTION

*Helicobacter pylori* is a Gram-negative, spiral-shaped, or rod-shaped microaerophilic bacterium with flagellar structures, primarily found in the mucus layer close to the surface of gastric epithelial cells (1, 2). The spiral shape and flagellar structure of *H. pylori* facilitate its movement and colonization in the gastric mucosa, thereby enhancing its invasiveness and pathogenicity (3). *H. pylori* infection is the main cause of atrophic gastritis, gastric ulcers, and duodenal ulcers, and if it is not eradicated in time, it may lead to the development of gastric cancer (4–6). In part due to antibiotic resistance, both first-line and rescue treatment regimens recommended in global guidelines for *H. pylori* infection face a failure rate of approximately 10%–30% (7). Studies have shown that globally, *H. pylori’*s resistance to clarithromycin, metronidazole, and levofloxacin has reached 27.2%, 39.7%, and 22.5%, respectively (8). Consequently, the treatment of *H. pylori* infection has become a global challenge to human health.

Bacterial biofilms are aggregates of bacteria encased in extracellular polymeric substances (EPS) that adhere to solid surfaces (9). In early studies, researchers used scanning electron microscopy to observe “dense accumulations of bacteria within an amorphous matrix” in the gastric tissue of people infected with *H. pylori* (10). *H. pylori* forms biofilm structures that separate bacteria from the external environment, reducing antibiotic penetration (11). Additionally, the coccoid form of *H. pylori* in biofilms and the upregulation of various efflux pump genes increase bacterial resistance to antibiotics (12). Recent studies have demonstrated that *H. pylori* biofilms enhance bacterial resistance to antibiotics, including amoxicillin, metronidazole, clarithromycin, and tetracycline (11–16). Investigation of *H. pylori* clinical isolates obtained from Indonesian patients demonstrated that strains with enhanced biofilm-forming capabilities exhibited significantly higher levels of multidrug resistance (17). A recent study demonstrated that pretreatment with N-acetylcysteine, which has been proven *in vitro* to inhibit biofilm formation and promote biofilm disruption, could effectively overcome antibiotic resistance and increase the eradication rate in patients with at least four previous *H. pylori* eradication failures (18). The presence of biofilms may enable persistent chronic *H. pylori* infection in the stomach, making it difficult to eradicate and significantly increasing the risk of gastric cancer due to long-term exposure to bacterial virulence factors (19).

The flagellar structure of *H. pylori*, consistent with most Gram-negative enteric bacteria, consists of three parts: the basal body, hook, and filament (3, 20, 21). Flagella play an important role in *H. pylori* colonization and persistent infection while also inducing the secretion of pro-inflammatory factors and enhancing inflammatory responses at the infection site (3). Recent reports suggest that *H. pylori* flagella are associated with biofilm formation. In *H. pylori* biofilm bacteria, genes related to flagellar structure and biosynthesis are upregulated compared to planktonic bacteria (22, 23). Studies have also shown that in flagellar structure gene mutant strains, bacterial biomass is significantly reduced in biofilm structures, suggesting that *H. pylori* flagellar structures contribute to biofilm formation (22–24).

In view of previous significant transcriptome studies revealing that multiple flagellar structural protein genes, including the *fliK* gene, were significantly higher in biofilm-forming *H. pylori* compared to non-biofilm *H. pylori* (22, 23). Our team’s recent research also found that a certain heat treatment, while inhibiting *H. pylori* biofilm formation, markedly decreased the expression level of the *fliK* gene (data not shown). Therefore, in this study, we investigate the role of the *fliK* gene in *H. pylori* biofilm formation. The *fliK* gene encodes the hook-length control protein, which acts as a “checkpoint control” by monitoring when the flagellar hook reaches its optimal length during structural assembly (25). Previous research has indicated a relationship between the *fliK* gene and bacterial biofilm formation in other species. For instance, in *Shewanella oneidensis MR-1*, deletion of the *fliK* gene affects the formation of three-dimensional biofilm structures (26). In *Vibrio cholerae*, biofilm formation is positively regulated by the anaerobic response regulator ArcA, which also downregulates *fliK* gene expression through direct and indirect pathways (27). To date, limited studies have investigated the functional role of the *fliK* gene in *H. pylori*. In terms of flagellar assembly regulation, mutation of the *fliK* gene upregulates the expression of RpoN-dependent flagellar genes (28, 29). During *H. pylori*’s adhesion to gastric epithelial cells, the *fliK* gene promotes the release of free, functional σ28, which initiates σ28-RNA polymerase, leading to significant upregulation of *cagA* in adherent *H. pylori* (30). However, no studies have explored the role of the *fliK* gene in *H. pylori* biofilm formation.

In this research, we examine changes in flagellar morphology and bacterial motility following *fliK* gene knockout in *H. pylori* strain NCTC 11637. Additionally, we reveal, for the first time, the gene’s impact on bacterial biofilm formation and alterations in extracellular matrix components.

## RESULTS

### Deletion of *fliK* alters flagellar structure in *H. pylori*

**Fig 1:**
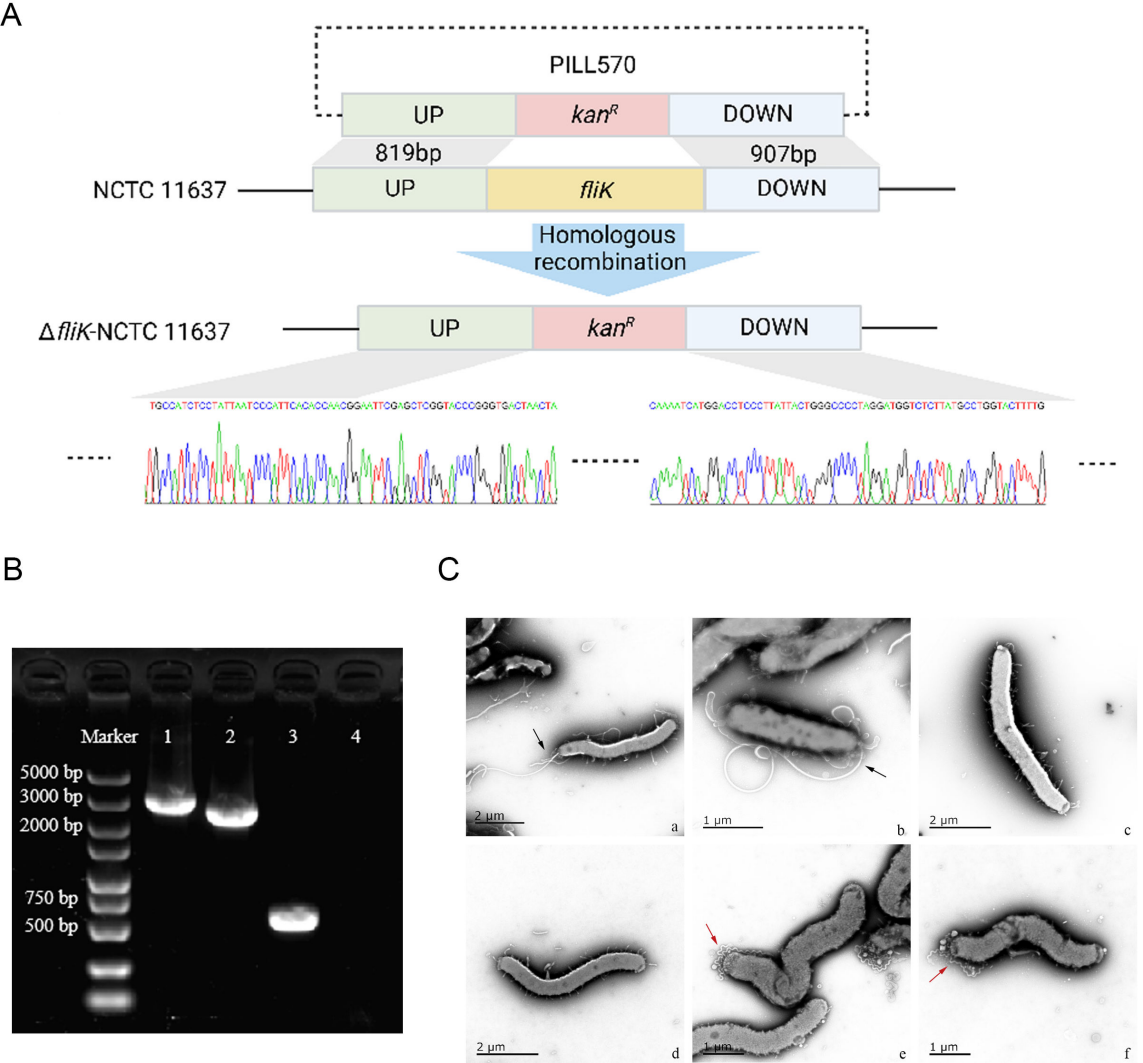
*fliK* gene knockout and the impact of *fliK* gene knockout on *H. pylori* flagella formation. (A) The *fliK* gene of *H. pylori* was knocked out by the homologous recombination method. (B) PCR verification of *fliK* gene deletion in NCTC 11637. Lane 1: PCR amplification of upstream homologous arm (UP) and *fliK* gene and downstream homologous arm (DOWN) (3,189 bp) using wild-type NCTC 11637 genomic DNA as template. Lane 2: PCR amplification of upstream homologous arm (UP) and *kan^R^* gene and downstream homologous arm (DOWN) (2,516 bp) using Δ*fliK*-NCTC 11637 genomic DNA as template. Lane 3: PCR amplification of *fliK* target gene (604 bp) using wild-type NCTC 11637 genomic DNA as template. Lane 4: PCR amplification of *fliK* target gene (no band) using Δ*fliK*-NCTC 11637 genomic DNA as template. (C) Transmission electron microscopy (TEM) observation of *H. pylori* flagella formation. (**a and b**) Wild-type NCTC 11637 strain; (**c and d**) *fliK* gene knockout strain (Δ*fliK*-NCTC 11637) lacked typical flagellar morphology; (**e and f**) Δ*fliK*-NCTC 11637 with polyhook structure of flagella. Black arrows indicate normal *H. pylori* flagella and red arrows indicate the polyhook structure of *H. pylori* flagella.

We used the recombinant plasmid PILL570 to knock out the *fliK* gene of *H. pylori* by homologous recombination. In short, we replaced the entire *fliK* gene with a gene that confers kanamycin resistance (*kan^R^*), creating a full deletion of *fliK* (Fig. 1A). PCR verification of the *fliK* deletion was performed using genomic DNA extracted from both wild-type and mutant strains. As shown in Fig. 1B, lane 1 represents the amplification of the wild-type fragment (upstream homologous arm + *fliK* gene + downstream homologous arm), while lane 2 shows the mutant fragment (upstream homologous arm + kanamycin resistance cassette + downstream homologous arm). The expected size difference between these fragments confirmed the successful replacement of the *fliK* gene with the kanamycin resistance cassette. To further confirm the successful deletion of the *fliK* gene, we designed primers to amplify *fliK* using PCR with genomic DNA from wild-type and Δ*fliK* strains as templates. As shown in lanes 3 and 4 of Fig. 1B, the *fliK* gene was present in the wild-type strain but absent in the deletion mutant, confirming successful knockout.

We then cultured wild-type and Δ*fliK* strains under microaerobic conditions for 3 days. Bacterial suspensions were adsorbed onto copper grids, negatively stained with phosphotungstic acid, and observed by transmission electron microscopy (TEM) to examine flagellar morphology before and after *fliK* deletion. The wild-type strain exhibited typical curved, long flagella of uniform thickness attached to one pole of the bacterial cell. However, the Δ*fliK* mutant exhibited polyhook structures or lacked typical flagellar morphology (Fig. 1C), suggesting that deletion of the *fliK* gene in *H. pylori* results in the formation of polyhook structures and prevents the assembly of normal flagellar filaments. Flagellar morphology was quantified from 20 randomly selected TEM images (magnification 1,000–2,000 times) for each strain (wild type and Δ*fliK*), with results shown in Table S2.

### Deletion of *fliK* impairs motility and growth of *H. pylori*

**Fig 2:**
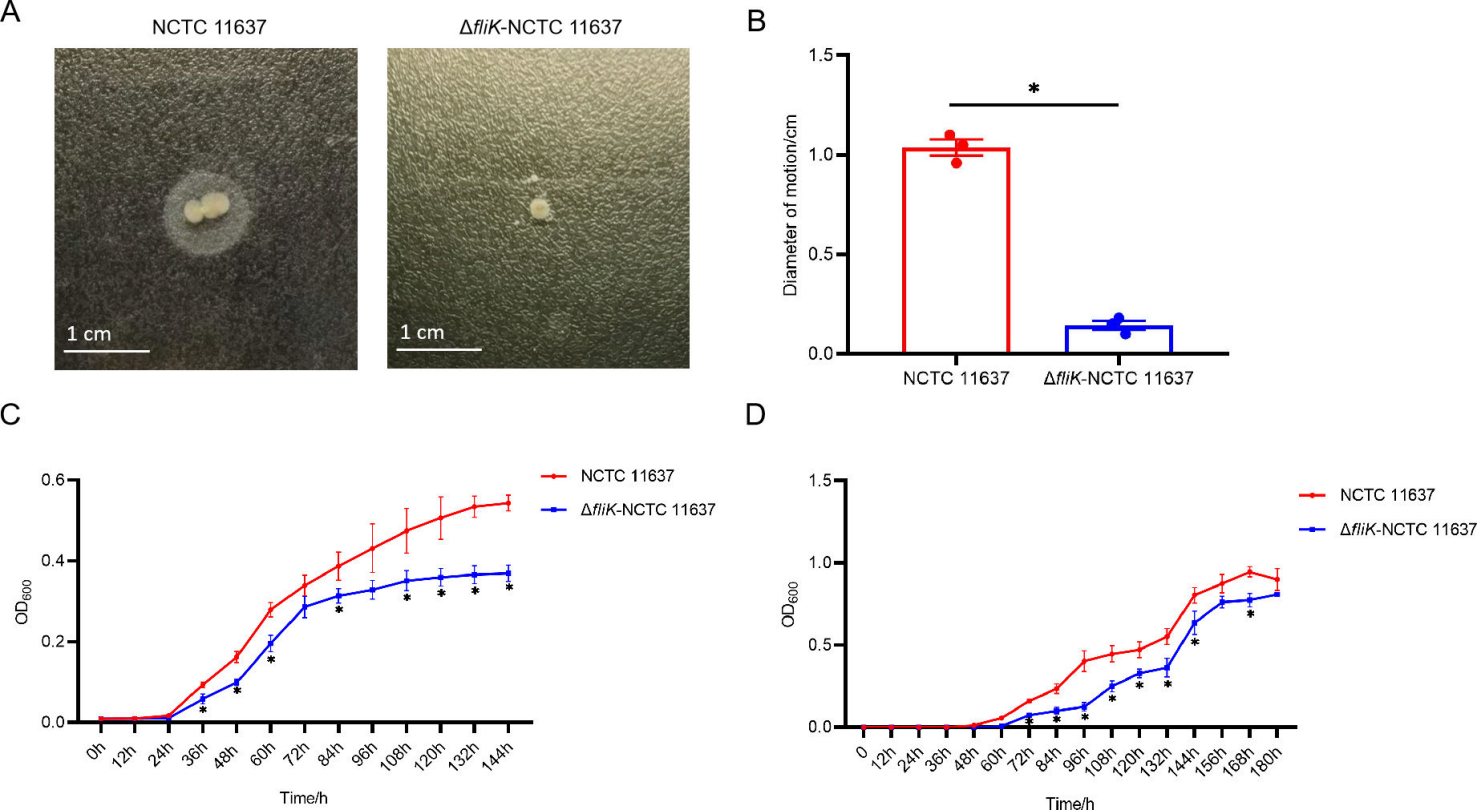
Impact of *fliK* gene deletion on *H. pylori* motility and growth ability. (A) Motility spread images of wild-type NCTC 11637 and Δ*fliK*-NCTC 11637 strains in a semi-solid medium. (B) Quantification of motility spread diameter for wild-type NCTC 11637 and Δ*fliK*-NCTC 11637 strains in semi-solid medium. (C) Growth curves of wild-type NCTC 11637 and Δ*fliK*-NCTC 11637 strains in liquid medium (*n* = 3, *P < 0.05). (D) Growth curves of wild-type NCTC 11637 and Δ*fliK*-NCTC 11637 strains on solid medium (*n* = 3, *P < 0.05).

We next investigated the effect of *fliK* deletion on *H. pylori* motility. *H. pylori* can utilize flagella to move through semi-solid media. We inoculated wild-type NCTC 11637 and Δ*fliK* strains into semi-solid agar and cultured under microaerobic conditions for 7 days before measuring the diameter of bacterial spread. As shown in Fig. 2A and B, the maximum spread diameter was 1.04 ± 0.06 cm for the wild-type strain compared to only 0.14 ± 0.03 cm for the Δ*fliK* mutant. Thus, deletion of *fliK* significantly impaired the motility of *H. pylori*.

To examine the impact of *fliK* deletion on bacterial growth, we cultured strains on solid and liquid media until the stationary phase, measuring bacterial density every 12 h to generate growth curves. In both solid and liquid culture conditions, loss of *fliK* resulted in slower growth of *H. pylori* (Fig. 2C and D). These results demonstrate that *fliK* deletion not only affects motility but also reduces the growth rate of *H. pylori* in various culture environments.

### *fliK* gene affects *H. pylori* adhesion to gastric epithelial cells

**Fig 3:**
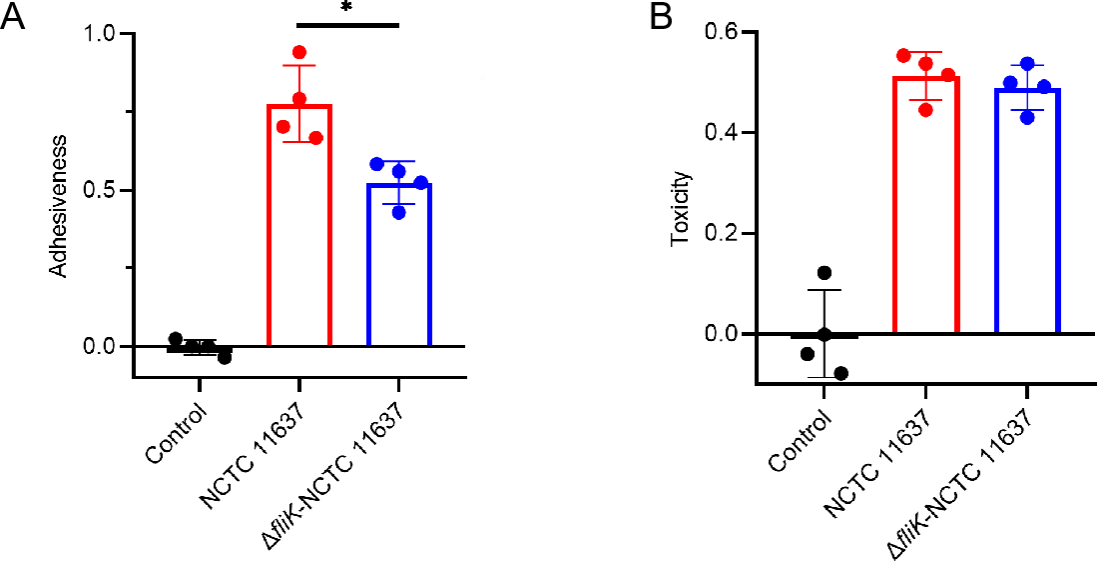
Impact of *fliK* gene deletion on *H. pylori* adhesion to gastric mucosal cells and vacuolating cytotoxicity. (A) Adhesion assay of *H. pylori* to GES-1 cells. GES-1 cells (1 × 10⁴/well) were co-cultured with wild-type NCTC 11637 or Δ*fliK*-NCTC 11637 strains (OD_600_ = 0.1) for 2 h. Bacterial adhesion was quantified by urease activity measurement (OD_540_). Data are presented as the relative increase in adhesion compared to the control group (cells without bacterial co-culture) (*n* = 4, *P < 0.05). (B) Vacuolating cytotoxicity assay of *H. pylori* on GES-1 cells. Cells were co-cultured with bacteria for 24 h, and vacuolation was assessed using a neutral red uptake assay (OD_550_). Data are presented as the relative increase in vacuolating cytotoxicity compared to the control group (cells without bacterial co-culture) (*n* = 4, *P < 0.05).

We further investigated the impact of *fliK* deletion on other phenotypes. First, we examined the effects on bacterial adhesion and vacuolating cytotoxicity to cells. Through co-culture of the two strains with gastric epithelial GES-1 cells, we found that deletion of *fliK* reduced bacterial adhesion to the cells (Fig. 3A), while there was no significant change in vacuolating cytotoxicity (Fig. 3B).

### Deletion of *fliK* reduces biofilm formation in *H. pylori*

Based on our previous findings, we further investigated the impact of *fliK* deletion on *H. pylori* biofilm formation. Bacteria were cultured in broth media supplemented with 7% fetal bovine serum (FBS) and incubated with shaking in 12-well plates for 3 days. Biofilm formation at the air-liquid interface was visualized by crystal violet staining. Additionally, we used SYTO9 fluorescent staining to visualize biofilms grown on nitrocellulose (NC) membranes for 3 days and observed them using laser confocal microscopy.

**Fig 4:**
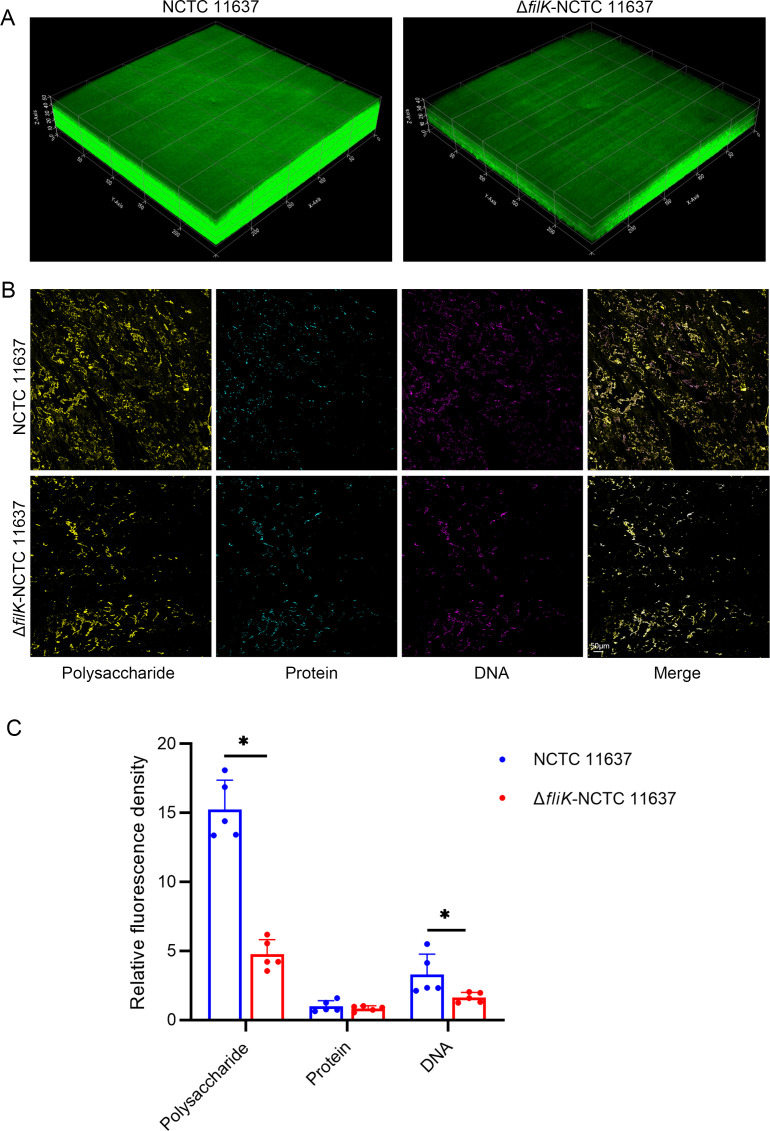
Impact of *fliK* gene deletion on *H. pylori* biofilm formation. (A) Laser confocal microscopy observation of biofilm morphology for wild-type NCTC 11637 and Δ*fliK*-NCTC 11637 strains. (B) Fluorescence staining of *H. pylori* biofilm components (polysaccharides, proteins, and DNA) (200×). The image shows the biofilm matrix of wild-type NCTC 11637 and Δ*fliK*-NCTC 11637 strains, where FITC-CoA labels polysaccharides, FilmTracer SYPRO Ruby labels proteins, and PI labels nucleic acids. (C) Quantitative analysis of fluorescence intensity for biofilm matrix components (polysaccharides, proteins, and DNA) in wild-type NCTC 11637 and Δ*fliK*-NCTC 11637 *H*. *pylori* strains (*n* = 5, *P < 0.05).

Crystal violet staining revealed that the Δ*fliK* mutant produced markedly less biofilm compared to the wild-type strain NCTC 11637 (Fig. S1). Consistently, fluorescence microscopy demonstrated that the wild-type NCTC 11637 strain formed biofilms approximately 30 µm thick with smooth, well-defined edges, whereas the Δ*fliK* mutant formed thinner biofilms of about 15 µm thickness, which were sparse and had rough edges (Fig. 4A). These results indicate that deletion of the *fliK* gene in *H. pylori* significantly reduces the bacterium’s ability to form biofilms.

### Deletion of *fliK* alters the composition of *H. pylori* biofilm matrix

We further investigated how *fliK* deletion affects the composition of the bacterial biofilm matrix. As the biofilms formed on NC membranes were too thick and dense for accurate fluorescence quantification, we analyzed biofilms grown on glass coverslips to examine changes in matrix composition.

Biofilms grown on coverslips were stained with different fluorescent dyes and observed under a laser confocal microscope. We randomly selected five fields of view for each sample to quantify fluorescence intensity. The results showed that deletion of the *fliK* gene in NCTC 11637 led to a significant reduction in polysaccharide and DNA components of the biofilm matrix, while protein content remained largely unchanged (Fig. 4B and C).

### Deletion of *fliK* does not alter antibiotic resistance in *H. pylori*

Finally, we investigated the impact of *fliK* deletion on bacterial antibiotic resistance. Using the E-test method, we examined changes in the minimum inhibitory concentrations (MICs) of commonly used antibiotics for *H. pylori* infection treatment. Our results showed that deletion of the *fliK* gene did not alter the resistance of the bacteria to commonly used antibiotics (clarithromycin, metronidazole, amoxicillin, tetracycline, and levofloxacin) (Table 1; Fig. S2).

**TABLE 1:** The minimum inhibitory concentrations of NCTC 11637 and Δ*fliK*-NCTC 11637

Antibiotic	MIC (μg/mL) for:	
	NCTC 11637	Δ*fliK*-NCTC 11637
Clarithromycin	0.064–0.094	0.064–0.094
Tetracycline	0.38–0.50	0.38–0.50
Levofloxacin	0.50–0.75	0.50–0.75
Amoxicillin	<0.016	<0.016
Metronidazole	>256	>256

## DISCUSSION

In this study, we first investigated the impact of *fliK* gene deletion on *H. pylori* flagellar structure. The FliK protein, encoded by the *fliK* gene, is known to act as a molecular ruler in bacteria (25). FliK consists of an N-terminal domain (FliKN) and a C-terminal domain (FliKC). FliKN interacts with the hook-cap protein FlgD and hook protein FlgE to measure flagellar hook length, while FliKC interacts with the export switch protein FlhB to control the transition from hook assembly to filament assembly (31–33). Previous studies on *fliK*, primarily conducted in *Salmonella*, have shown that when the flagellar hook reaches a length of 55 nm, FliKC undergoes a conformational change, binds to FlhBC, and activates the type III protein export apparatus to switch from hook-type to filament-type specificity (34). Similar studies on FliK have demonstrated that insertion or deletion of one amino acid in FliKN results in an increase or decrease in hook length by approximately 0.2 nm (35). However, the regulatory role of *fliK* and FliK protein on flagella may vary among different bacteria. In *Salmonella*, mutations in different domains of FliK affect flagellar hook length; in *Campylobacter jejuni*, *fliK* deletion mutants exhibit unusual flagellar structures such as “polyhooks”; and in *Bacillus thuringiensis* strain Bt407, *fliK* deletion mutants show no flagellar structures under atomic force microscopy (36–38). A transcriptomic study on *H. pylori fliK* insertion mutants suggested that *fliK* mutation affects the transcription of almost all flagellar regulatory factors in *H. pylori* (28). As for *fliK* mutants of *H. pylori* have been reported, *fliK* mutants exhibited abnormally elongated hook structures lacking filaments and led to dysregulated expression of flagellar genes (25, 39). Our observations are consistent with these findings, as we observed typical sheathed flagellar structures in wild-type *H. pylori* NCTC 11637 under transmission electron microscopy, while deletion of the *fliK* gene exhibited polyhook structures or lacked typical flagellar morphology.

We then assessed changes in *H. pylori* motility after *fliK* deletion and found that the Δ*fliK* strain exhibited significantly reduced motility compared to the wild-type strain in semi-solid media. Previous reports have highlighted the relationship between flagella and motility. *H. pylori* generates motility through 2–6 polar flagella, which are composed of FlaA and FlaB flagellins and precisely regulated by a hierarchical gene expression system controlled by σ54 (RpoN) and σ28 (FliA), this flagella-mediated motility system enables efficient swimming in highly viscous gastric mucus (21, 40). What’s more, Cheng-Yen Kao et al. reported that mutations in *csrA* and *rpoN* genes led to loss of flagellar structure and reduced bacterial motility (41). The deletion of the *flgV* gene, which in *H. pylori* forms a high-torque motor ring in flagellar assembly, results in reduced flagella and motility defects (42). The deletion of *flhF* in *H. pylori* resulted in reduced motility, hypoflagellation, and the improper localization of flagella to non-polar sites (43).

Previous studies have demonstrated that *H. pylori* is capable of forming biofilms *in vitro* and biofilm-like structures *in vivo* (10). Biofilm formation involves steps including adhesion, assembly, maturation, and dispersion (44). *H. pylori* flagella have been found to participate in various stages of biofilm formation. Flagellar motility is involved in *H. pylori* adhesion, with studies showing that strains with flagellar structure but no motility (Fla+Mot-) exhibit significantly reduced attachment to AGS cells compared to wild-type strains (23). During biofilm assembly, the absence of normal flagellar structure in *H. pylori* has been found to slow biofilm assembly, and flagellar filaments have been shown to promote inter-bacterial connections, maintaining biofilm integrity on both biological and non-biological surfaces (23, 45). Our observations using fluorescence confocal microscopy confirmed that defects in *H. pylori* flagellar structure significantly affect biofilm formation.

We further analyzed the composition of *H. pylori* biofilms grown on glass coverslips and found that *fliK* deletion significantly reduced polysaccharide and DNA components. The integrity of flagellar structure and function has been reported to influence changes in bacterial biofilm EPS components. In *Pseudomonas aeruginosa* and *Pseudomonas putida*, the transcriptional regulator FleQ, involved in flagellar gene expression, controls extracellular polysaccharide synthesis in a c-di-GMP-dependent manner, affecting the expression of biofilm EPS components (46, 47). In *Bacillus subtilis*, EpsE interacts with FliG to inhibit flagellar rotation while also acting as an EPS biosynthetic enzyme, jointly promoting biofilm formation (48). However, there are currently no reports on *H. pylori* biofilm EPS components in the literature.

*H. pylori* pathogenesis in the gastroduodenal environment is attributed to its adhesion ability and virulence factors. We examined the effect of *fliK* deletion on *H. pylori* adhesion to gastric mucosal cells and vacuolating cytotoxicity. Our results showed reduced bacterial adhesion but no significant change in vacuolating cytotoxicity. *H. pylori* adhesion is primarily achieved through bacterial adhesins binding to host cells, such as blood group antigen-binding adhesin and sialic acid-binding adhesin binding to Lewis antigens and sialylated Lewis antigens on the host gastric mucosa, thus not only helping bacteria to colonize the stomach for a long time but also effectively resisting gastric acid erosion and evading the host immune response (49, 50). Previous studies have found that mutations in *H. pylori* flagellar regulatory genes not only affect motility but also significantly reduce adhesion to host cells (51). It has also been proposed that mutations in flagella-related genes (e.g., *fliI*) affect the expression of *H. pylori* adhesins (52, 53). Although we do not have direct evidence that flagella promote *H. pylori* adhesion to gastric epithelial cells, we speculate that the *fliK* gene may be involved in regulating *H. pylori* adhesin synthesis, thus explaining the reduced adhesion to gastric mucosal cells upon *fliK* deletion.

*H. pylori*’s vacuolating cytotoxicity is directly related to its vacuolating cytotoxin (VacA). VacA has been demonstrated to function as a secreted multifunctional toxin that can assist bacterial colonization and survival. The toxin’s ability to cause cellular vacuolation is maintained in culture supernatants and purified preparations without requiring constant bacterial attachment, thus may indicate that its activity is independent of bacterial adhesion (54, 55). Studies have shown that VacA induces the formation of intracellular vacuoles in late-stage *H. pylori*-infected cells, providing a suitable environment for *H. pylori* survival and facilitating persistent infection and toxicity (56). Furthermore, VacA often works in conjunction with the *cagA* pathogenicity island (cag PAI), enabling CagA to exert cytotoxic effects on the host through the type IV secretion system (57). *vacA* transcription may be regulated by factors such as low pH, iron concentration, salt concentration, and bacterial contact with host cells (58–61). However, there are currently no reports on the impact of flagellar genes on vacuolating cytotoxicity, and thus no evidence to suggest that *fliK* deletion would alter bacterial vacuolating cytotoxicity to gastric mucosal cells.

We also observed that *H. pylori fliK* deletion significantly slowed bacterial growth. Previous studies have indicated that bacterial flagella and proliferation are mutually influential, with flagellated bacterial cell division affecting flagellar regulon expression, and flagellar structure assembly and maturation regulating bacterial division (62, 63). In *Escherichia coli*, flagellar transcriptional activator (*flhD*) gene mutants exhibit faster division rates and smaller cell sizes compared to wild-type strains (64). Therefore, we speculate that the flagellar assembly process in *H. pylori* may also influence bacterial proliferation, though the specific mechanisms require further investigation.

Finally, our results showed that *fliK* deletion did not alter *H. pylori* antibiotic resistance. *H. pylori* antibiotic resistance mechanisms involve multiple molecular and biological pathways, including gene mutations, upregulation of drug efflux pumps, and biofilm formation (65). Point mutations in *H. pylori* resistance genes directly affect antibiotic targets or bacterial metabolic pathways, leading to antibiotic ineffectiveness, such as *rdxA* mutations causing metronidazole resistance and *gyrA* mutations leading to levofloxacin resistance (66, 67). Efflux pumps are multidrug transporters on bacterial cell membranes that export antibacterial drugs from the bacterial cytoplasm, reducing intracellular antibiotic concentrations (68). Biofilms not only form effective and non-specific barriers preventing penetration of various drugs and reducing direct antimicrobial effects but also promote horizontal gene transfer and overexpression of efflux pumps involved in resistance (69, 70). Currently, there are no reports on the relationship between *H. pylori* flagellar-related genes and resistance loci or efflux pump function. Regarding the impact of biofilm formation on *H. pylori* resistance, we believe that the *fliK* gene may affect bacterial resistance by influencing biofilm formation, but not affecting planktonic bacteria prior to biofilm formation. Transmission electron microscopy results also suggest that *fliK* deletion did not disrupt the integrity of bacterial cell membranes and cell walls.

A limitation of the present study is the lack of genetic complementation of the Δ*fliK* mutant. However, several lines of evidence support the specificity of our *fliK* deletion. First, we employed homologous recombination to precisely delete the *fliK* coding sequence, taking care to preserve the adjacent gene sequences. Second, the phenotype of altered flagellar structure we observed in the Δ*fliK* mutant highly resembles those reported in *H. pylori* and other bacteria such as *Salmonella* and *Campylobacter jejuni* (36, 39, 71, 72). Therefore, the observed phenotypic changes can be attributed specifically to the loss of *fliK* function rather than the disruption of other genes in the operon.

**Fig 5:**
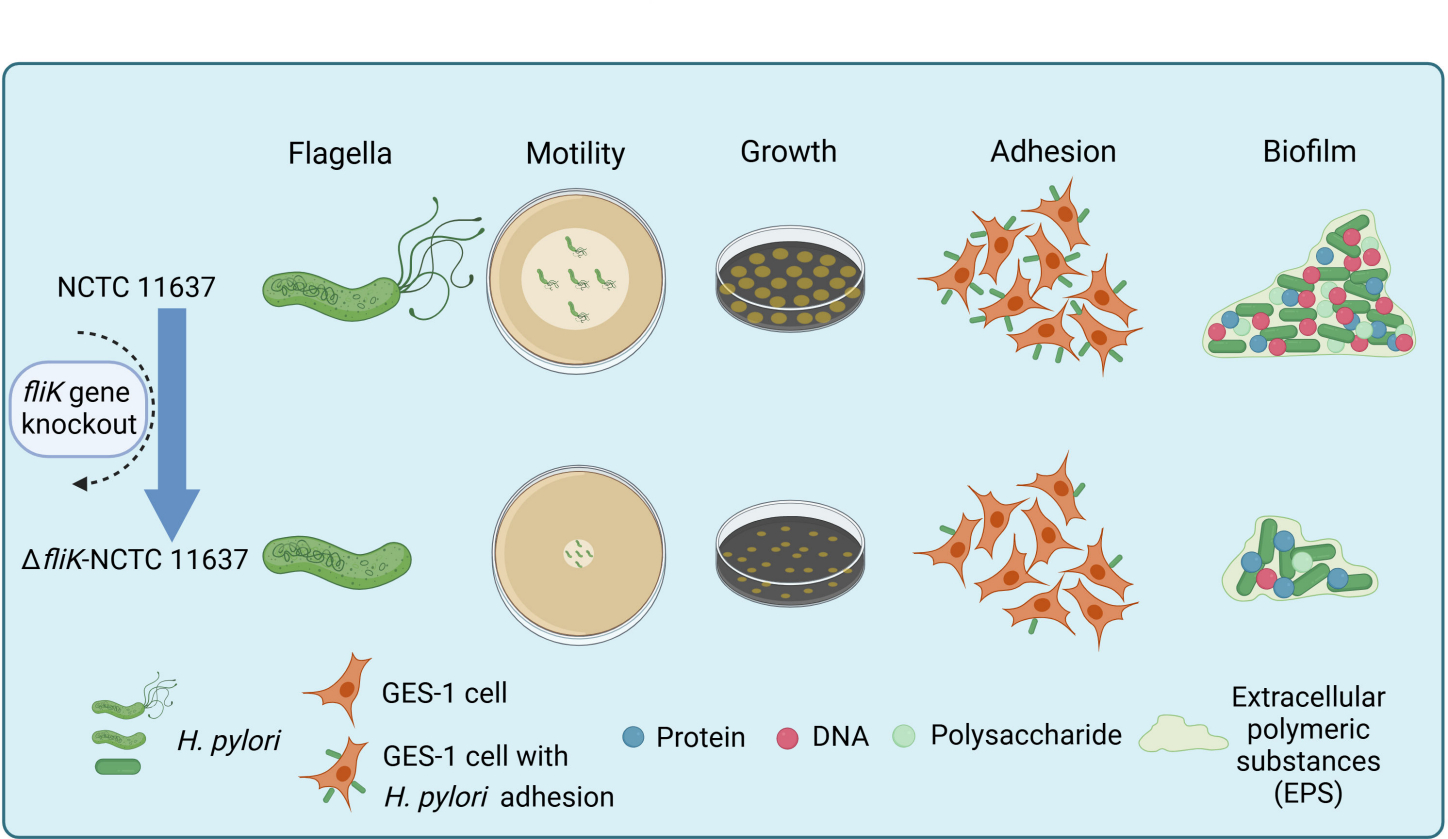
Impact of *fliK* knockout on *H. pylori* NCTC 11637. Comparison of phenotypes between the Δ*fliK* mutant strain (Δ*fliK*-NCTC 11637) and the wild-type strain (NCTC 11637), indicating abnormal flagella morphology, reduced motility, slower growth rate, weakened adhesion capability, and decreased biofilm formation.

Further research is needed to elucidate the regulatory mechanisms of the *fliK* gene on *H. pylori* biofilm formation. In conclusion, our study investigated the impact of the *fliK* gene on *H. pylori* phenotypes and demonstrated that *fliK* deficiency significantly affects bacterial biofilm formation, specifically reducing biofilm components such as polysaccharides and DNA (Fig. 5). These findings provide new insights for research on *H. pylori* biofilms and potential therapeutic strategies.

## MATERIALS AND METHODS

### Bacterial strains and culture conditions

*H. pylori* type strain NCTC 11637 used in this study was identified and authenticated using 16S rRNA gene sequencing. *H. pylori* was cultured under microaerobic conditions (5% O_2_, 10% CO_2_, and 85% N_2_) at 37°C for 3 days. Solid culture medium consisted of Karmali Agar Base (CM0935, Oxoid, UK) supplemented with 5% sterile defibrinated sheep blood. Liquid culture medium was brain heart infusion supplemented with 10% FBS. Semi-solid medium was prepared by adding 0.3% agar to the liquid medium.

### Construction of *fliK* gene knockout strain

The *fliK* gene knockout strain of *H. pylori* was constructed according to a previously published protocol (73), with detailed experimental procedures provided in Table S1. The knockout plasmid PILL570 containing a kanamycin resistance gene was kindly provided by Professor Yundong Sun from Shandong University. Briefly, we amplified the upstream and downstream homologous arms of the *fliK* gene and inserted these two sequences into PILL570. The constructed recombinant plasmid was then transformed into bacterial cells, replacing the target *fliK* gene with the kanamycin resistance gene. Finally, we designed primers (Table S1) to verify the successful deletion of the *fliK* gene by PCR amplification using genomic DNA from wild-type and *fliK* knockout strains as templates.

### Transmission electron microscopy observation of *H. pylori* flagella

Wild-type NCTC 11637 and *fliK* knockout strains were collected in PBS and adjusted to a concentration of 1 OD_600_ (1 × 10^8^ CFU). Carbon fiber membranes were floated on the bacterial suspension for 8 min to allow bacterial adsorption. Excess liquid was removed with filter paper, and 2% phosphotungstic acid stain was added for 2 min. After removing excess stains, the samples were air-dried naturally. The stained carbon fiber membranes were observed under TEM to examine *H. pylori* flagellar morphology.

### Motility assay

Wild-type NCTC 11637 and *fliK* knockout strains were collected in PBS and adjusted to a concentration of 1 OD_600_ 2 µL of bacterial suspension was injected into a prepared semi-solid medium and cultured under microaerobic conditions for 7 days to observe bacterial motility in the medium.

### *H. pylori* growth curve

*H. pylori* was cultured in solid and liquid media. For solid media, five single colony clones were randomly scraped from each plate every 12 h and collected in 1 mL PBS to measure bacterial concentration until growth reached the plateau phase. For liquid media, bacterial concentration was measured every 12 h until growth reached the plateau phase.

### *H. pylori* adhesion and vacuolating cytotoxicity assay

Human gastric mucosal GES-1 cells in the logarithmic growth phase were seeded in 96-well plates (1 × 10^4^/well) and cultured overnight. Each well was inoculated with 200 µL of wild-type NCTC 11637 or *fliK* knockout strain suspension (0.1 OD_600_) or 200 µL of blank medium as control. For the adhesion assay, after 2 h co-culture, the supernatant was discarded, and wells were washed with PBS three times. One hundred microliters of urea reagent was added to each well and incubated at 37°C for 2 h. Absorbance was measured at 540 nm using a microplate reader. For vacuolating cytotoxicity assay, after 24 h co-culture, the supernatant was discarded, and 100 µL of 0.05% neutral red stain was added for 5 min. After washing with PBS three times, 100 µL of 0.04% hydrochloric acid–ethanol was added, and absorbance was measured at 550 nm. The change in absorbance values of experimental groups compared to the control group was calculated to reflect changes in adhesion and vacuolating cytotoxicity.

### *H. pylori* biofilm and component detection

Biofilm formation was assessed using crystal violet staining and fluorescence microscopy with confocal laser scanning microscopy. For crystal violet staining (74), logarithmic-phase *H. pylori* were collected and suspended in broth media supplemented with 7% FBS to a final concentration of 5 × 10^5^ CFU/mL. The bacterial suspension (1 mL per well) was cultured in 12-well plates at 120 rpm for 3 days. After removing the medium, the wells were washed three times with PBS, followed by staining with 500 µL of 1% (wt/vol) crystal violet solution for 30 min. The crystal violet dye was subsequently dissolved in an 80% ethanol–20% acetone solution, and the absorbance was measured at OD_580_. For fluorescence staining of biofilms, autoclaved 1 cm × 1 cm NC membranes were placed on solid medium surfaces. Ten microliters of bacterial suspension (0.5 OD_600_) was spotted onto the NC membrane, air-dried, and cultured inverted for 3 days. Biofilms were stained using LIVE/DEAD BacLight Bacterial Viability Kits (Invitrogen, USA), with SYTO9 dye labeling all bacteria with green fluorescence. After washing with sterile PBS to remove planktonic bacteria, 200 µL of SYTO9 was added and incubated in the dark for 20 min. Excess dye was removed by washing with PBS three times. The NC membrane was placed on a glass slide, mounted with an anti-fade reagent, and observed under a confocal microscope.

For biofilm component detection, autoclaved coverslips were placed in 6-well plates containing 2 mL of liquid medium. Ten microliters of bacterial suspension (1 OD_600_) was added and cultured on a shaker at 120 rpm for 3 days to allow biofilm growth on the coverslips. Coverslips were then fixed with 200 µL glutaraldehyde at 4°C for 1.5 h and washed with PBS three times. Components were stained using FilmTracer SYPRO Ruby biofilm matrix stain for proteins, FITC-CoA for polysaccharides, and propidium iodide for nucleic acids. After staining, coverslips were mounted with anti-fade reagent and observed under a confocal microscope. Fluorescence intensity was quantified using Image J software.

### *H. pylori* antibiotic resistance testing

The E-test (Epsilometer test) method was used to determine *H. pylori*’s resistance to amoxicillin, levofloxacin, metronidazole, clarithromycin, and tetracycline. The MIC was determined by reading the value at the intersection of the inhibition zone formed on the culture medium with the E-test strip.

### Statistical analyses

All statistical analyses were performed using SPSS software (version 22.0; IBM Corp., Armonk, NY, USA). Data were presented as mean ± standard deviation, and differences between the two groups were analyzed using Student’s *t*-test. Statistical significance was defined as *P* < 0.05.

## SUPPLEMENTAL MATERIAL

Supplemental figures msphere.00018-25-s0001.pdf Fig. S1 and S2.

Supplemental tables msphere.00018-25-s0002.xls Tables S1 and S2.

ASM does not own the copyrights to Supplemental Material that may be linked to, or accessed through, an article. The authors have granted ASM a non-exclusive, world-wide license to publish the Supplemental Material files. Please contact the corresponding author directly for reuse.
