# Supplementary material for: The impact of the flagellar protein gene fliK on Helicobacter pylori biofilm formation
Source: mSphere. 2025 Mar 21;10(4):e00018-25. doi: 10.1128/msphere.00018-25 (PMC12039246; doi:10.1128/msphere.00018-25)
Supplement: Supplemental figures — Fig. S1 and S2. [file msphere.00018-25-s0001.pdf]

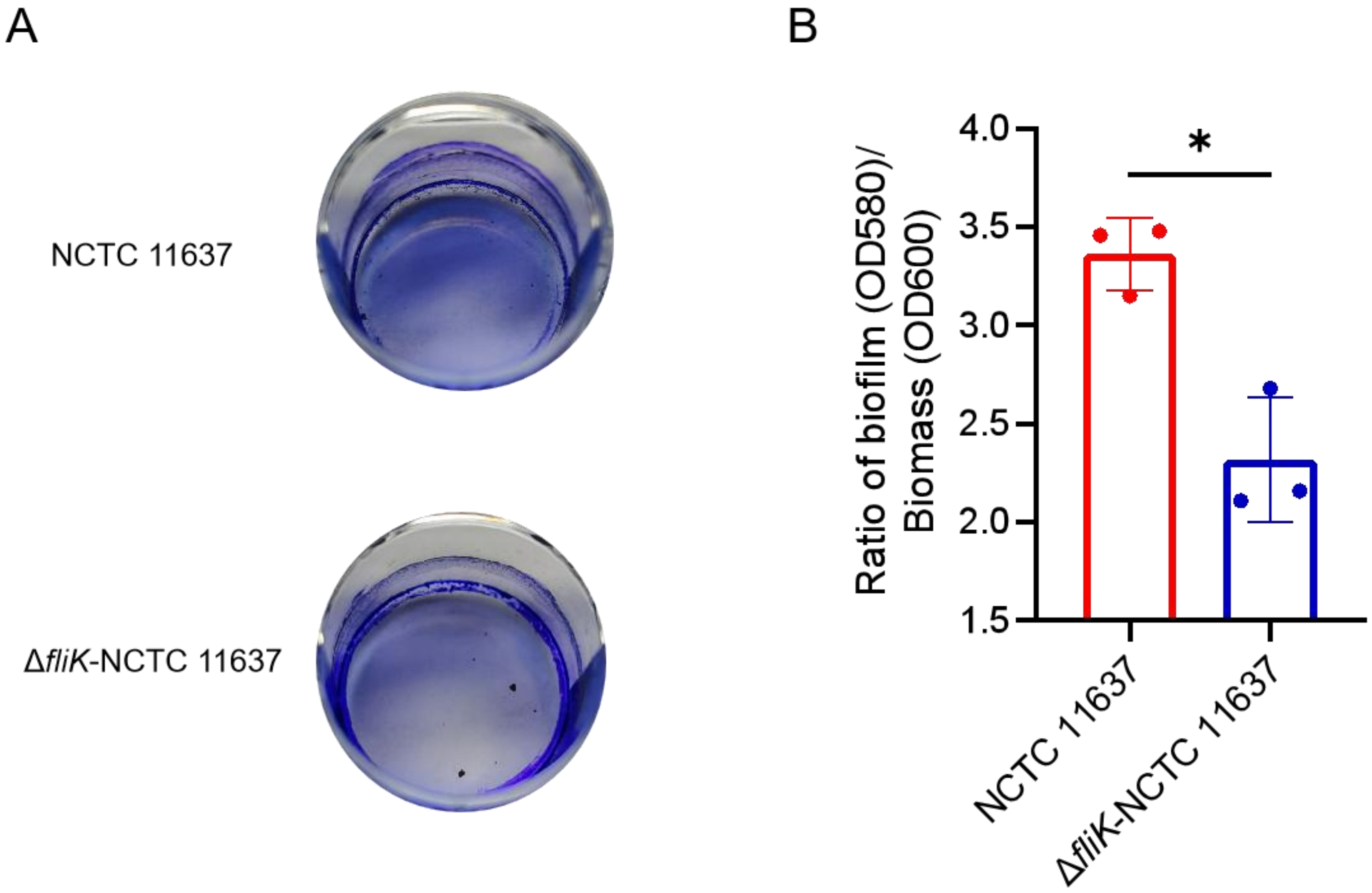

Figure S1. Effects of *fliK* deletion on *H. pylori* biofilm formation. **A.** Crystal violet staining of biofilms formed by wild-type *H. pylori* NCTC 11637 (WT) and Δ*fliK*-NCTC 11637 after 3 days of cultivation in 24-well plates. **B.** Changes of bioflm-to-biomass ratios of NCTC 11637 and Δ*fliK*-NCTC 11637. Quantification of biofilm formation was measured by the crystal violet dye dissolved in an 80% ethanol-20% acetone solution, and the absorbance was measured at OD_{580}. Biomass was the final bacterial concentration. (n=3, *$p<0.05$)

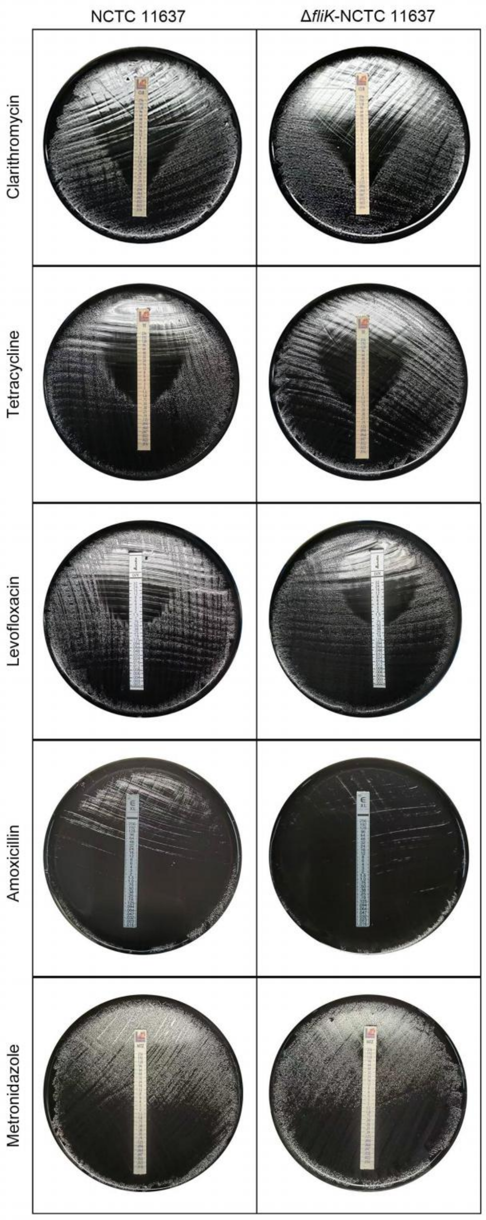

Figure S2. Antibiotic susceptibility results for wild-type NCTC 11637 and Δ*fliK*-NCTC 11637 *H. pylori* strains.
